# An Innovative Prone Position Using a Body-Shape Plaster Bed and Skull Traction for Posterior Cervical Spine Fracture Surgeries

**DOI:** 10.3389/fsurg.2022.649421

**Published:** 2022-03-10

**Authors:** Zhiyu Ding, Yijun Ren, Hongqing Cao, Yuezhan Li, Shijie Chen, Jinglei Miao, Jinsong Li

**Affiliations:** ^1^Department of Spine Surgery, The Third Xiangya Hospital, Central South University, Changsha, China; ^2^Department of Neurology, Xiangya Hospital, Central South University, Changsha, China

**Keywords:** innovative prone position, cervical spine surgery, body-shape plaster bed, skull traction, posterior approach

## Abstract

**Background:**

An innovative prone cervical spine surgical position using a body-shape plaster bed with skull traction (BSPST) was compared with the traditional prone surgical position with horseshoe headrests.

**Methods:**

A total of 47 patients, undergoing posterior cervical spine surgery for cervical spine fracture, were retrospectively classified into two groups, the BSPST group (*n* = 24) and the traditional group (*n* = 23), and underwent a posterior instrumented fusion with or without decompression. Multiple indicators were used to evaluate the advantages of the BSPST compared with the traditional position.

**Results:**

All the operations went smoothly. The mean recovery rate was 56.30% in the BSPST group and 48.55% in the traditional group (*p* = 0.454), with no significant difference. The intraoperative blood loss (177.5 ml vs. 439.1 ml, *p* = 0.003) and the total incidence of complications (8.3 vs. 47.8%, *p* = 0.004) were significantly less in the BSPST group than in the traditional group. In addition, the BSPST position provided a greater comfort level for the operators and allowed convenient intraoperative radiography.

**Conclusions:**

This is the first study to describe a combined body-shape plaster bed and skull traction as an innovative cervical spine-prone surgical position that is simple, safe, and stable, intraoperative traction direction adjustable, reproducible, and economical for posterior cervical spine fracture surgery, and potentially other cervical and upper dorsal spine surgeries in the prone position. Additionally, this position provides the surgeons with a comfortable surgical field and can be easily achieved in most orthopedic operation rooms.

## Introduction

The prone position is widely used globally for posterior cervical and dorsal spine surgeries (1). To date, the traditional posterior approach to the surgical stabilization of the head and the cervical spine is usually achieved by the horseshoe headrest (2). This system, however, has many shortcomings, such as being unavailable and unstable for position adjustment during surgery. Additionally, inappropriate pressure from the horseshoe headrest over the eyeballs and facial skin may cause damage to patients, especially during long-duration cervical spine surgery in the prone position (3–5).

According to our experience, a good patient surgical position is essential for smooth surgery and should be safe, stable, and adjustable during surgery to reduce postoperative complications. The authors present a modification of the prone surgical position for posterior cervical spine surgeries using a cervical tong for skull traction and a body-shape plaster bed for fixing the patients' bodies. We named this innovative prone surgical position the “body-shape plaster bed with skull traction” (BSPST). This innovative system using a body-shape plaster bed avoids a localized pressure associated with the horseshoe headrest and allows free access to anesthetists for better endotracheal tube management. Additionally, the body-shape plaster bed and skull traction support a reliable stable fixation at any time during surgery and can also be available for intraoperative position adjustment and radiography.

Based on our previous experiences in posterior cervical and dorsal spine surgeries, we used the BSPST position for 24 patients with unstable cervical spines; i.e., traumatic cervical cord injury caused by cervical spine fracture, undergoing surgical treatment in the prone position. There have been no previous reports of the combination of a body-shape plaster bed and cervical tong used in cervical spine surgery. To evaluate the effectiveness and safety of the BSPST position in posterior cervical spine surgeries, comparisons in perioperative events including positioning time, surgical time, intraoperative blood loss, complications, neurological improvement, and comfort of surgeons between cases using the BSPST position and traditional prone surgical position were performed.

## Materials and Methods

### Patient Population

A total of 205 patients, who underwent prone position cervical spine surgery from June 2017 to February 2018 in our institute, were included and were retrospectively reviewed. All procedures performed in the studies involving human participants were in accordance with the ethical standards of the institutional and national research committee (The IRB of the Third Xiangya Hospital, Central South University. Reference number: No. 2019-S036) and with the 1964 Helsinki declaration and its later amendments or comparable ethical standards. All the participants and any identifiable individuals consented to the publication of his/her images. After excluding cases with the degenerative cervical syndrome (*n* = 62), degenerative cervical spinal stenosis (*n* = 20), ossification of the cervical spine yellow ligament (*n* = 9), ossification of the cervical spine posterior longitudinal ligament (*n* = 33), intraspinal occupying lesion of the cervical spine (*n* = 26), and other cervical spine diseases (*n* = 7), a total of 47 patients with cervical spine fracture were included and divided into a BSPST group (using the BSPST position, *n* = 24) and a traditional position group (using horseshoe headrest position, *n* = 23) as shown in Table 1. None of the included patients had other diseases related to the cervical spine fracture, and coagulation function test results were normal for every patient. All patients received regular outpatient visits or telephone follow-up every 3 months, and the final follow-up was defined as the 12-months postoperative follow-up. Informed consent was obtained from all individual participants included in the study.

**Table 1:** General preoperative information in two groups.

	**Traditional position**	**BSPST position**	***p*** **value**
	**(***n*** = 23)**	**(***n*** = 24)**	
Age (year)	52.09 ± 12.76	52.67 ± 15.89	0.891
Gander (M\F)	21\2	20\4	0.413
Diabetes (n)	3	5	0.478
Hypertension (n)	4	4	0.947
BMI	23.22 ± 4.30	23.30 ± 4.05	0.949
**Surgery levels of cervical spine of patients**			0.885
2 levels	3	5	
3 levels	8	6	
4 levels	7	9	
5 levels	4	3	
6 levels	1	1	

### The Body-Shape Plaster Bed

Six different sizes (XS, S, M, L, XL, and XXL) of the body-shape plaster bed was made by our doctors and can be used repeatedly. We used the bandage and cotton pad to cover the body-shape plaster bed surface every time before surgery so that the bed can keep clean. Doctors must choose the most suitable body-shape plaster bed for each patient before surgery. According to our experience, the distance between the patients' forehead and lower jaw must match the diameter of the plaster bed head-ring, while the total length and width of the plaster bed have no strict requirements because of the use of soft cotton pad and surgical drapes.

### The Innovative BSPST Position

**Figure 1:**
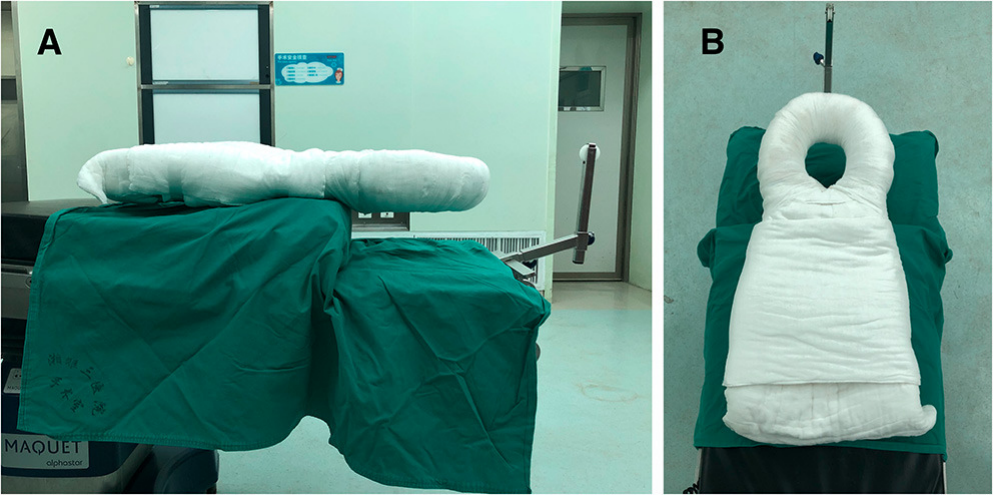
The assembly of traction system set on a conventional operating table and the body-shape plaster bed as seen from different positions **(A,B)**. The body-shape plaster bed with skull traction (BSPST) position system can provide enough space for managing anesthesia tube.

**Figure 2:**
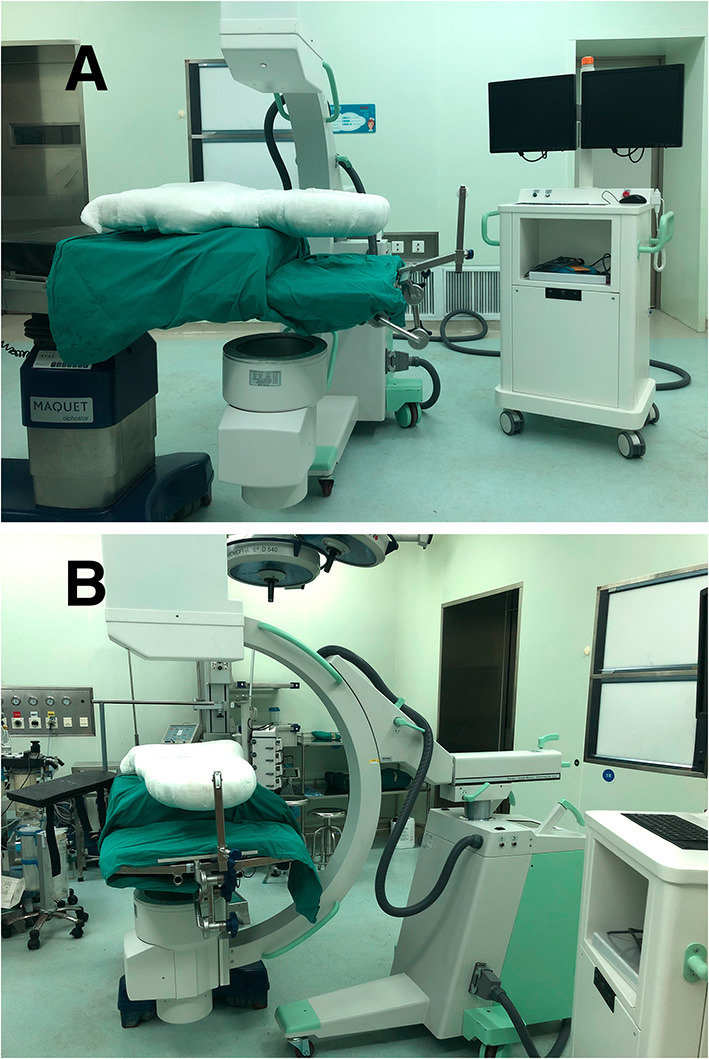
The BSPST system and C-arm digital radiography machine from different positions **(A,B)**, which can make anterior-posterior interoperative radiography available.

**Figure 3:**
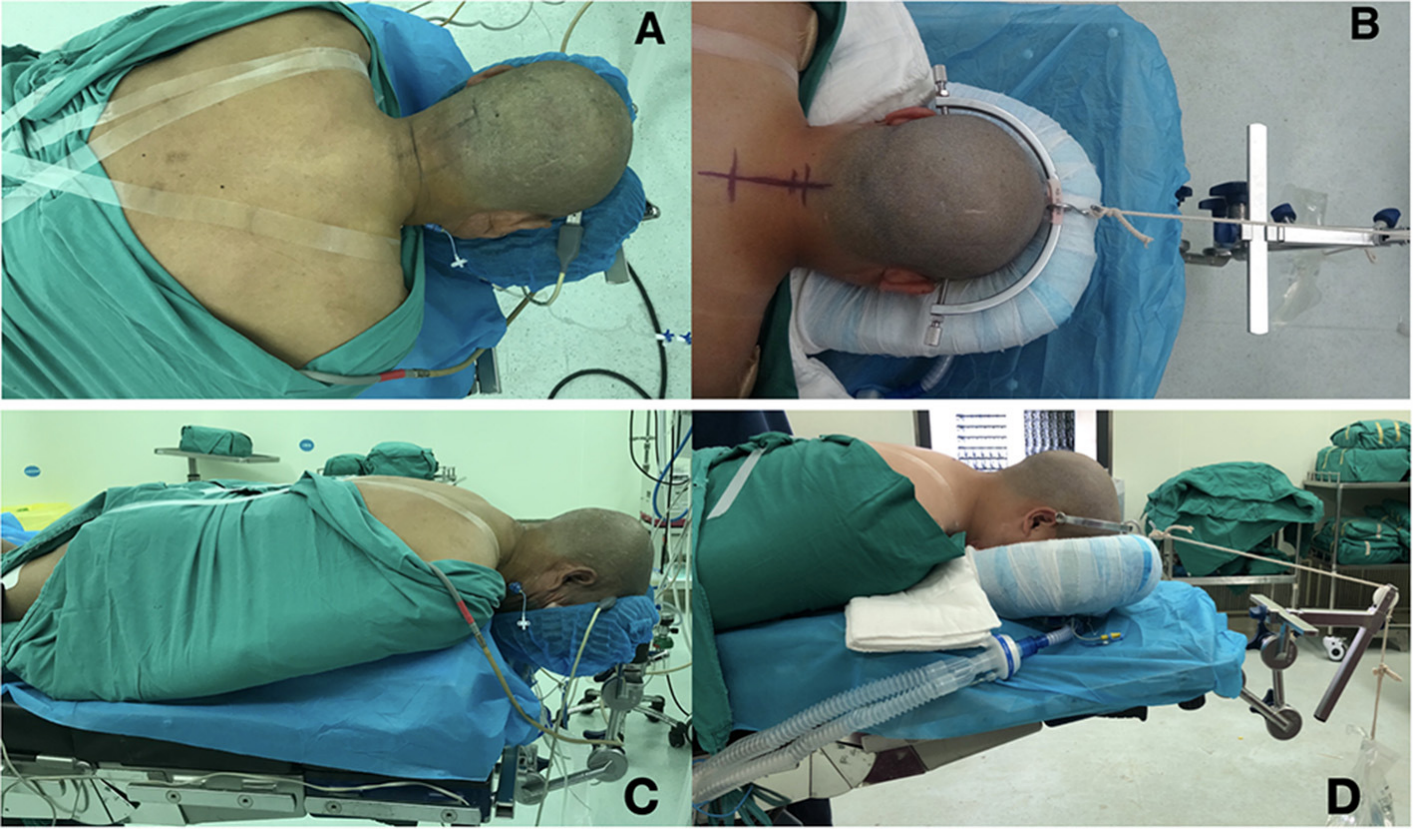
The patients undergoing surgical treatment in the prone position. The patients in the traditional position with a horseshoe headrest **(A,C)** and in the BSPST position with a cervical tong for skull traction and body-shape plaster bed for fixation **(B,D)** as seen from a vertical and horizontal angle. The BSPST position can provide enough space for managing the anesthesia tube and reliable intraoperative steadiness with the usage of body-shape plaster bed and skull traction.

All surgeries were performed by a senior surgeon with the same standard. The patients were placed in the supine position after general anesthesia on a surgical transfer trolley, and after the body-shape plaster bed was buckled onto the patient's head and chest (see Supplementary File 1), the whole body and the body-shape plaster bed were turned over together and placed in the prone position; then, an anesthesia tube was attached and a surgical drape or bandage was used to bind the patient, body-shape plaster bed, and operation table together to ensure stability (see Supplementary File 2). The skull traction was assembled in the appropriate direction and placed on a conventional operating table (here an Alphastar bed, MAQUET GmbH & Co. KG, Sweden) as shown in Figures 1, 2. Subsequently, surgeons and assistants can adjust the posture of the cervical spine and head by putting a soft cotton pad between the patient's shoulder and the body-shape plaster bed before surgery. Together, the body-shape plaster bed and traction system provide stability to the patient during the adjustment of the operating table and can provide enough space for intraoperative anesthesia tube management (Figure 3 and Supplementary Files 2, 3). Additionally, this system allows the intraoperative adjustment of the traction direction and weight by the traction system. At the end of the surgery, the patient was turned supine, and the tong was removed (see Supplementary File 4). The pin sites were dressed with band-aids.

### The Limit of Usage

Patients with severe cervical and thoracic kyphosis.

### Data Statistics and Clinical Assessments

**Figure 4:**
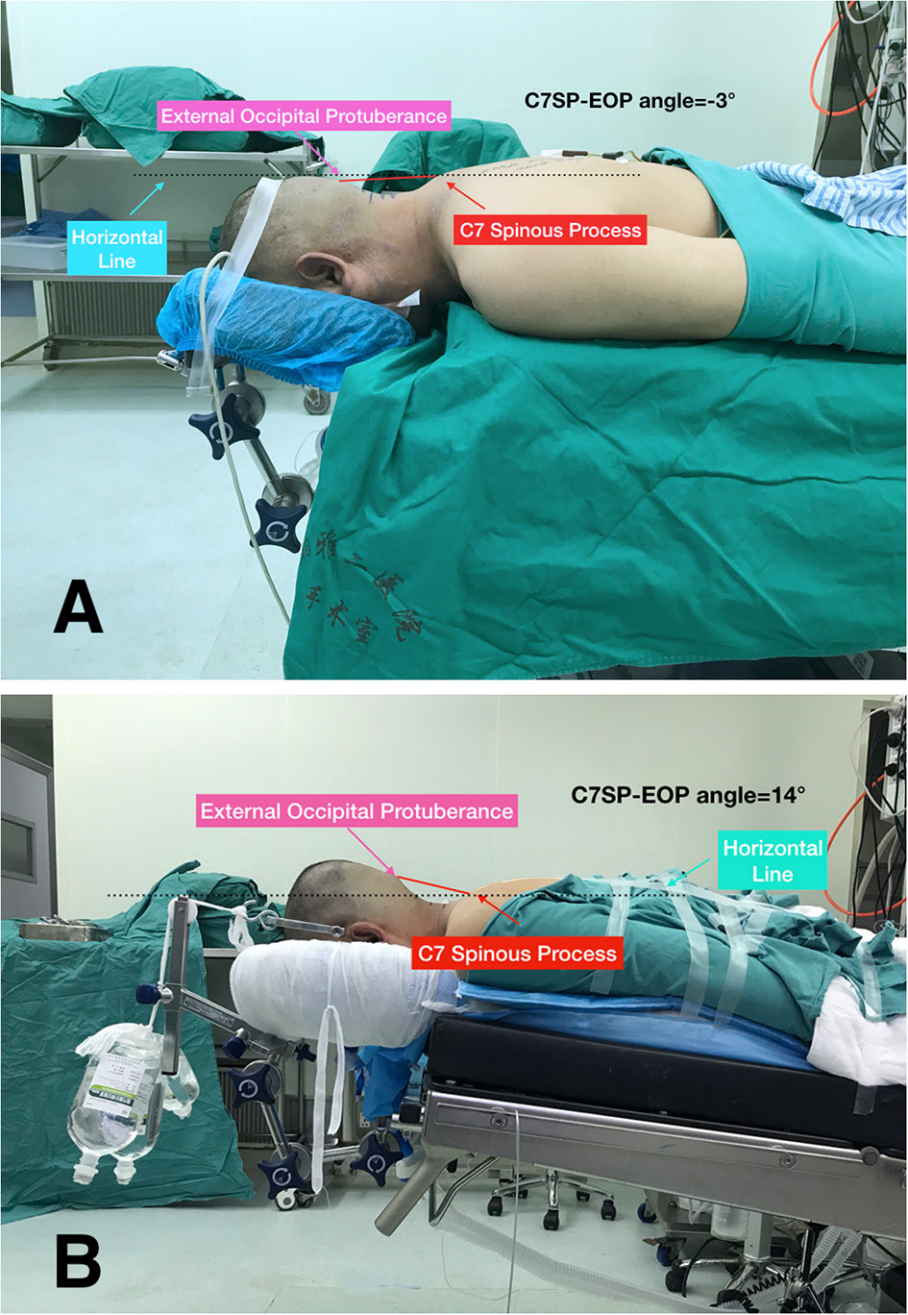
The angle between the C7 spinous process-external occipital protuberance line and horizontal line (C7SP-EOP angle) was defined as a system to evaluate the degree of surgeon comfort during the operation. The traditional position is assessed at level 1 **(A)**, while the BSPST position at level 4 **(B)**.

All the patients underwent radiologic examinations, including CT and MRI of the cervical spine before surgery. The modified Japanese Orthopedic Association (JOA) scores were used to assess neurological function, and the neurological recovery rate was calculated as = (final JOA - preoperative JOA) / (11 - preoperative JOA) × 100%. The neurological recovery rate of 75–100% was designated as excellent; 50–74%, good; 25–49%, fair; and <25%, poor. According to our experience in cervical spine surgeries and cervical spine anatomy, we described an assessment of intraoperative surgeon comfort. The angle between the C7 spinous process-external occipital protuberance line and the horizontal line (C7SP-EOP angle) was categorized into four levels from −5° to 15° (Figure 4). Level 4 was defined as the most comfortable position for surgeons with a C7SP-EOP angle between 10° and 15°, while Level 1 was defined as a difficult process to finish the surgery for surgeons with a C7SP-EOP angle between −5° and 0°. Intraoperative blood loss, operation time, and positioning time (the time required after induction of anesthesia until positioning the patient prone on the operating table), perioperative complications, C7SP-EOP angle, and possibility for intraoperative radiography were recorded and compared between the two groups.

### Statistical Analysis

All the data were analyzed by PASW Statistics 18.0 (SPSS Inc., Chicago, IL, USA). Intragroup or intergroup comparisons were performed by independent samples *t*-test or Pearson's χ2 test, and data were presented as means and SD unless otherwise indicated. *P* < 0.01 were defined as statistically significant.

## Results

The differences in general preoperative information between the two groups were not significant, as shown in Table 1. A total of 169 segments (traditional position group, *n* = 84; BSPST group, *n* = 85) were involved. The distribution of the surgical levels was not significantly different between the two groups (*p* = 0.885).

As shown in Table 2, the intraoperative blood loss in the BSPST group was significantly less than that in the traditional position group (177.5 ml vs. 439.1 ml, *p* = 0.003), while the operation time (3.755 h vs. 4.400 h, *p* = 0.144) and position time (16.25 min vs. 15.96 min, *p* = 0.184) have no significant differences. Although the mean JOA scores in the BSPST group and traditional position group significantly increased at the final follow-up, differences in the preoperative JOA score (7.875 vs. 7.913, *p* = 0.979), 6-months follow-up JOA score (12.46 vs. 11.09, *p* = 0.353), final JOA score (12.46 vs. 11.09, *p* = 0.353), and neurological recovery rate (56.30 vs. 48.55%, *p* = 0.454) were not significant. There were six patients in the traditional position group, but no patients in the BSPST group, who experienced facial skin necrosis (*p* = 0.007), and these patients completely recovered after a consultation in the department of dermatology with regular dressing changes for 1 week. Only two cases in the traditional position group received conjunctival and corneal abrasions but recovered without treatment within several days (*p* = 0.140). The leakage of cerebrospinal fluid occurred with one patient in the traditional position group, but no patient in the BSPST group, and this patient experienced an incision healing without infection (*p* = 0.302). Both groups had one patient with C5 palsy after the operation that healed on its own (*p* = 0.976). The traditional position group had one patient with wound infection (*p* = 0.302), and this patient was completely healed after regular dressing changes and the use of intravenous antibiotics within one postoperative week. There was only one patient with a cervical tong pin site complication in the BSPST position group (*p* = 0.322). The patient was bleeding under galea aponeurotica because of the use of low molecular weight heparin in the intensive care unit, and the blood was gradually absorbed after stopping the use of low molecular weight heparin. The total incidence of perioperative complications in the BSPST group was significantly less than that in the traditional position group (*p* = 0.004).

**Table 2:** Comparisons in outcomes and complications between two groups.

	**Traditional position (*n* = 23)**	**BSPST position (*n* = 24)**	***p*** **value**
Operation time (h)	4.400 ± 1.752	3.755 ± 1.136	0.144
Positioning time (min)	15.960 ± 6.832	16.250 ± 6.835	0.184
Intraoperative blood loss (ml)	439.1 ± 369.0	177.5 ± 105.2	0.003*
**JOA score**			
Before surgery	7.913 ± 5.017	7.875 ± 4.675	0.979
6-months follow-up	9.736 ± 5.268	11.040 ± 4.506	0.366
12-months follow-up	11.090 ± 5.468	12.460 ± 4.520	0.353
Neurological recovery rate (%)	48.550 ± 35.770	56.300 ± 34.690	0.454
**Grading of neurological recovery rate (** ***n*** **)**			0.176
Excellent	7	9	
Good	2	7	
Fair	7	3	
Poor	7	5	
**Complication, number of patients**			
Face skin necrosis	6	0	**0.007^#^**
Conjunctival and corneal abrasions	2	0	0.140
POVL	0	0	——
Injury to spinal cord (Leakage of CSF)	1	0	0.302
C5 palsy	1	1	0.976
Wound infection	1	0	0.302
Cervical tong pin sites complications	0	1	0.322
Difficult access to anesthesia tubes	0	0	——
Total incidence of complications (%)	47.8	8.3	**0.004^§^**

*JOA, Japanese Orthopedic Association*.

* *Statistically different from the intraoperative blood loss (p < 0.01)*.

# *Statistically different from the face skin necrosis cases (p < 0.01)*.

§ *Statistically different from the total incidence of complications (p < 0.01)*.

There were 20 patients in the BSPST position group but only one patient in the traditional position group who were assessed at level 4 or level 3 in the evaluation system for the degree of comfort of the surgeon during the operation as shown in Table 3 (*p* < 0.01). The BSPST position allowed for both anterior-posterior and lateral intraoperative radiography, while the traditional position did not allow them (*p* < 0.01).

**Table 3:** C7SP-EOP angle (comfort level for surgeons) and intro-op radiography.

	**Traditional position**	**BSPST position**	***p*** **value**
	**(*n* = 23)**	**(*n* = 24)**	
Comfort level for surgeons			0*
Level 4	0	15	
Level 3	1	5	
Level 2	10	4	
Level 1	12	0	
Intro-op radiography			
A-p (unable\able)	23\0	0\24	**0^#^**
Lateral (unable\able)	0\23	0\24	——

* *Statistically different from the comfort level of surgeons (p < 0.01)*.

# *Statistically different from the anterior-posterior intro-operation radiography (p < 0.01)*.

## Discussion

According to lots of research, many complications have been reported due to the disadvantages of the traditional posterior approach position, such as postoperative visual loss (POVL), skin necrosis, venous air embolism, etc. (3, 6). These complications may have serious consequences to the patients, but researchers have only sporadically attempted to modify the traditional prone surgical position.

Improper pressure over the eyeballs and facial skin for a long time is a common cause of visual loss and skin necrosis. Several studies have discussed a postoperative vision loss due to the prone position (3). They conclude that the inappropriate pressure from the horseshoe headrest led to direct pressure over the eyeball, which may cause intraocular pressure and visual loss (7, 8). In addition, there have been many documented cases of facial pressure sores and ischemic orbital compartments related to the prone position and horseshoe headrest (3). It has been reported that the long-term localized pressure on the face in the prone position is, on average, below 30 mmHg, but can be higher than 50 mmHg in certain areas, such as the chin and forehead above the supraorbital ridge, which may cause facial edema and pressure sores (9–11).

According to our experience, surgeons may need intraoperative readjustment of patients' positions for a better surgical field. The traditional prone position has no fixation of the patients' head, body, and operating table, so it is impossible to ensure the stability of patients when adjusting the operating table and the traction direction during the operation, which may lead to a respiratory passage compression and asphyxia. Additionally, Kadam et al. (2) proposed a modified prone position for posterior cervical spine surgeries using a cervical tong for traction and two lateral brace attachments on an operating table, which can avoid a localized pressure over the eyeballs and the face skin associated with the horseshoe headrest. However, this modified prone position has the inability to intraoperatively readjust the position and tilt the table beyond 30° to either side.

Immobilization by a cervical collar to protect the patient from secondary damage is a standard procedure in patients with cervical spine trauma (12, 13). However, more studies have pointed out that applying a cervical collar, in general, will cause an immense three-dimensional movement, and extrication collars can result in abnormal movement within the upper cervical spine in the presence of a severe injury (14–16). We believe that an absolute restriction of the cervical spine cannot be only achieved by the cervical collar during preoperative positioning and may cause secondary dislocation in those with spinal cord injury, especially in the presence of a dissociative injury (17).

The BSPST position can protect the facial skin and the eyes from skin necrosis and ocular complications with the use of protective macromolecular material. The body-shape plaster bed can decrease the vertical direct pressure by distributing the pressure equally across the facial skin, while the round head holder has no direct contact with the patients' eyes. In our analysis of the traditional and BSPST positions, we identified 10 patients (47.8%) with postoperative complications in the traditional position group and three patients (8.3%) with postoperative complications in the BSPST group (*p* = 0.004). This result showed that the incidence of postoperative complications was relatively high when cervical spine surgery incorporated a traditional prone surgical position compared with the BSPST prone surgical position.

As for the adjustment of the surgical position, the BSPST position can maintain a stable position even when the table exceeds 35° to either side. Additionally, the traction direction can be intraoperatively adjusted to expose the operation fields for obese and short-neck patients. This method also allows the patients to be stably positioned in the reverse Trendelenburg's position because the traction can balance with part of the patients' own gravity (see Supplementary File 3), which can reduce venous congestion and bleeding, as well as reduce orbital pressure to diminish the occurrence of postoperative vision loss (6, 18). It was obvious in our research that only one patient in the traditional position group, but 20 patients in the BSPST group, provided the surgeon the comfort levels of 1 and 2 (*p* < 0.01), and intraoperative blood loss in the BSPST position group was significantly less than that in the traditional position group (*p* = 0.003). Since the plaster bed extends to the abdominal region, we think that the part of the plaster bed in contact with the abdomen may compress the soft tissues and increase the abdominal pressure; to avoid this situation, we put cotton and soft gel pads around the contact part to reduce the direct pressure on the abdomen. All these results indicated that the BSPST position may provide the surgeons with a more comfortable surgical position and reduce intraoperative blood loss.

To maintain safety during the preoperative positioning, the surgeon and assistants can create a situation in which the patient and body-shape plaster bed stay together so that the patients' head, whole cervical spine, and body can turn around at the same time by using the body-shape plaster bed. The BSPST position also facilitated an easy access to the anesthesia tube, which could be removed from either side below the body-shape plaster bed (Figures 1, 2 and Supplementary File 2).

**Figure 5:**
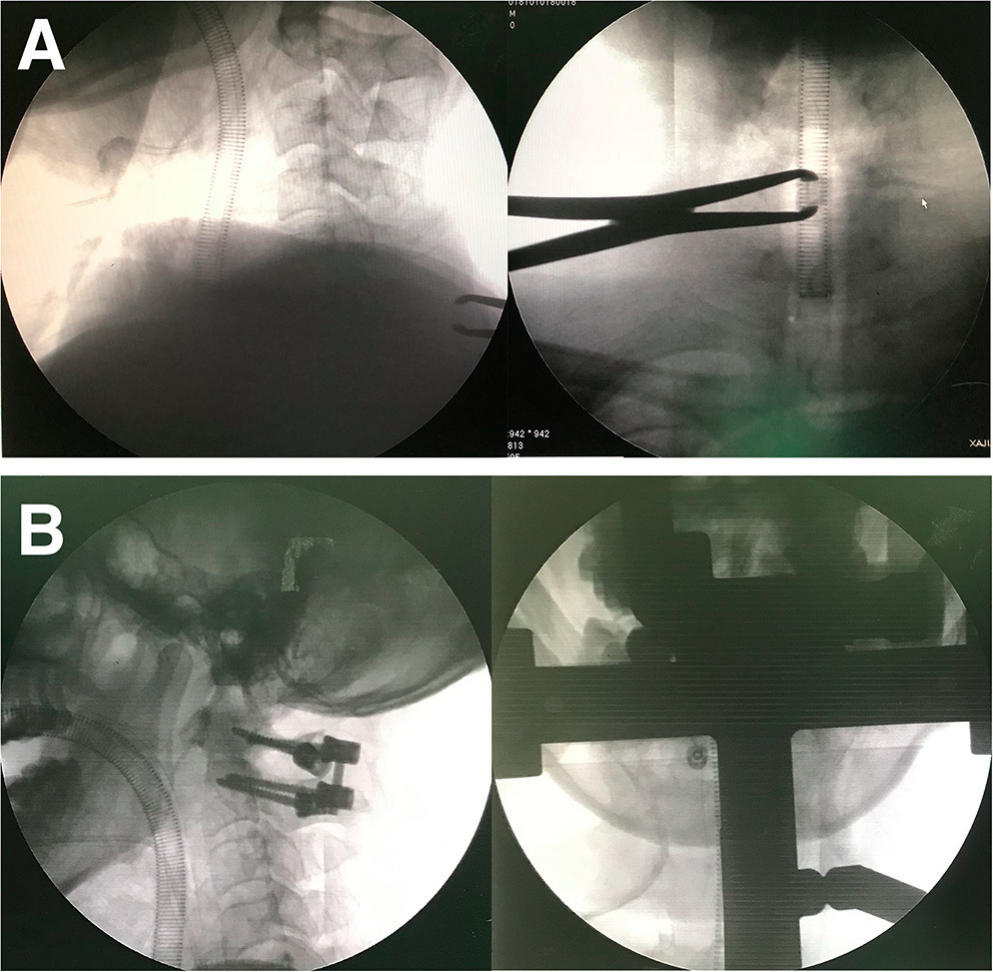
Anterior-posterior intro-operation radiography is available in BSPST position benefit from X-ray penetrable characteristics of plaster **(A,B)**.

Intraoperative radiography is necessary for spine surgery, especially cervical spine surgery, and it can help surgeons conform to surgical segments and guide, as well as conform to pedicle screw placement (19). However, the anterior-posterior interoperative radiography is unavailable in the traditional position because of the material of the headrest, which may create difficulties for the surgeons (Figures 2, 5). Although carbon fiber headrest is a good choice, most hospitals and patients in developing countries cannot afford it. The BSPST position system is X-ray penetrable, easy to assemble and inexpensive, and can be acceptable for patients in many hospitals in developing countries compared with other innovations of prone position for cervical spine surgeries (20–22).

For patients with obesity, the soft tissues of the cervicothoracic transition can cause wrinkling of the cervical skin incision. To solve this problem, we put a soft cotton pad between the shoulder and the plaster bed to slightly change the kyphosis of the cervical spine. We also use a shoulder strap or tape to pull the fat tissue to the tail end at the same time, and the skinfold can be solved when fixed with the head.

The positioning time was 16.250 ± 6.835 min in the BSPST group and 15.960 ± 6.832 min in the traditional group, which were not significantly different (*p* = 0.144). Although cervical tong application may appear to require additional time, it is a relatively quick procedure, and the time consumed is well-compensated by the reduced time required to reduce skin necrosis and ocular complications. In addition, the BSPST position can be used not only in cervical spine fracture surgeries but also in other posterior cervical spine surgeries.

In this research and our clinical work, the BSPST position and traditional position almost have the same effect in prone cervical spine decompression and fixation surgeries to treat cervical spine fracture. However, the body-shape plaster bed may provide a better choice to some hospitals in developing countries because of the lower prices.

All techniques have downsides, and there are still several limitations of this study and of the BSPST position. First, retrospective results from a single-center should be prospectively verified by multicenter and randomized controlled studies. Second, the patient sample was relatively small, and the follow-up was relatively short in this study. Besides, this position is not available for patients with advanced deformity, and has some disadvantages that are directly related to the prone position. In addition, the measurement of the C7SP-EOP angle may show significant deviations in patients with obesity due to the thick fat tissue around the neck and back. Finally, further studies are required to conclusively establish the efficacy and the safety of the BSPST position to put it into use and improve upon it.

## Conclusion

This is the first study to describe a combined body-shape plaster bed and skull traction as an innovative prone surgical position that has characteristics of simple construction, safe and stable, intraoperative traction direction adjustable, reproducible, and economical for posterior cervical spine fracture surgery and potentially other cervical and upper dorsal spine surgeries in the prone position. Additionally, this position can be easily achieved in most operating rooms and provides a comfortable surgical field for surgeons. However, further studies are required to conclusively establish the efficacy and safety of this innovative method.

## Data Availability Statement

The raw data supporting the conclusions of this article will be made available by the authors, without undue reservation.

## Ethics Statement

The studies involving human participants were reviewed and approved by the IRB of the Third Xiangya Hospital, Central South University. The patients/participants provided their written informed consent to participate in this study.

## Author Contributions

Material preparation was performed by ZD and YR, data collection and analysis were performed by HC, YL, JM, and SC. The first draft of the manuscript was written by ZD and JL and all the authors commented on previous versions of the manuscript. All the authors read, approved the final manuscript, and contributed to the study's conception and design.

## Funding

This study was partially supported by the National Natural Science Foundation of China (Grant Nos. 81502331 and 81772866) and the Natural Science Foundation of Hunan Province (Grant Nos. 2016JJ3176, 2018JJ2602, and 2018JJ2617). The study supporters played no role in the study design, collection, analysis, and interpretation of data, in the writing of the manuscript, and in the decision to submit the manuscript for publication.

## Conflict of Interest

The authors declare that the research was conducted in the absence of any commercial or financial relationships that could be construed as a potential conflict of interest.

## Publisher's Note

All claims expressed in this article are solely those of the authors and do not necessarily represent those of their affiliated organizations, or those of the publisher, the editors and the reviewers. Any product that may be evaluated in this article, or claim that may be made by its manufacturer, is not guaranteed or endorsed by the publisher.

## Supplementary Material

Supplementary File 1 The model was placed in the supine position after “general anesthesia” on a surgical transfer trolley, and the body-shape plaster bed was buckled onto the model's head and chest (This is an illustrative video, and not an actual patient).

Supplementary File 2 The model's whole body and the body-shape plaster bed were turned over together by the surgeon and the assistants and placed in the prone position; then, an anesthesia tube was attached. The head, whole cervical spine, and body can turn around at the same time with the use of the body-shape plaster bed (This is an illustrative video and not an actual patient).

Supplementary File 3 The body-shape plaster bed and skull traction support a reliable stable fixation at any time during surgery, even in reverse Trendelenburg position (This is an illustrative video and not an actual patient). The C7SP-EOP angle is 11.3° in the BSPST position during the surgery which provides the most comfortable position for surgeons (Practical surgery).

Supplementary File 4 At the end of the surgery, the model and the body-shape plaster bed were turned supine, the tong and body-shape plaster bed were removed (This is an illustrative video and not an actual patient).
